# Retrospective analysis of free temporoparietal fascial flap for defect reconstruction of the hand and the distal upper extremity

**DOI:** 10.1007/s00402-020-03635-9

**Published:** 2020-11-01

**Authors:** Wibke Müller-Seubert, Raymund E. Horch, Vanessa Franziska Schmidt, Ingo Ludolph, Marweh Schmitz, Andreas Arkudas

**Affiliations:** 1 Department of Plastic and Hand Surgery of the Friedrich-Alexander-University Erlangen-Nürnberg (FAU), Krankenhausstr. 12, 91054 Erlangen, Germany; 2 Present Address: Department of Radiology, University Hospital LMU Munich, Marchioninistr. 15, 81377 Munich, Germany

**Keywords:** Temporoparietal fascial flap, Hand, Upper extremity, Reconstruction

## Abstract

**Introduction:**

Soft tissue reconstruction of the hand and distal upper extremity is challenging to preserve the function of the hand as good as possible. Therefore, a thin flap has been shown to be useful. In this retrospective study, we aimed to show the use of the free temporoparietal fascial flap in soft tissue reconstruction of the hand and distal upper extremity.

**Methods:**

We analysed the outcome of free temporoparietal fascial flaps that were used between the years 2007and 2016 at our institution. Major and minor complications, defect location and donor site morbidity were the main fields of interest.

**Results:**

14 patients received a free temporoparietal fascial flap for soft tissue reconstruction of the distal upper extremity. Minor complications were noted in three patients and major complications in two patients. Total flap necrosis occurred in one patient.

**Conclusion:**

The free temporoparietal fascial flap is a useful tool in reconstructive surgery of the hand and the distal upper extremity with a low donor site morbidity and moderate rates of major and minor complications.

## Introduction:

The temporoparietal fascial flap (TPFF) was first described by Golovine in 1898 [1, 2]. To this day it is still a useful tool in reconstructive surgery [3]. It can be used as a pedicled flap to cover defects of the scalp, midface, mandible and oral cavity [4]. As a free flap it can cover defects whenever a very thin layer of tissue is needed. The long pedicle and the low donor site complications as well as the inconspicuous scar are the main advantages of this flap [1, 2]. These characteristics result in a good functional and aesthetic outcome especially when the defect is located at the hand. Furthermore, harvesting the flap and exposing the recipient vessels is possible simultaneously, if transplanting the TPFF to the upper extremity [5].

The anatomy of the TPFF has been described earlier [4, 6–9]. The temporalis fascia (TPF) is a thin, highly vascularized tissue that covers the temporalis muscle. It has a superficial and a deep layer, in between connective tissue and fat tissue. The TPF is mainly supplied by the superficial temporal artery (STA). Recent studies have shown that 88% of the TPF get their blood supply from the STA, nine percent are supplied by the posterior auricular artery, and three percent by the occipital artery [10]. During harvesting the flap, two nerves are in close proximity to the temporalis fascia: the frontal branch of the facial nerve is located under the temporalis fascia, the auriculotemporal nerve is 5 mm close to the superficial temporal artery until 1,5 cm above the helix [11]. Flap thickness is usually between 2 and 4 mm and a TPFF can be harvested up to a size of 14 × 17 cm^2^ [7, 11]. A further advantage of the flap is the possibility of harvesting a second combined pedicled flap: the temporalis muscle [7].

To present its suitable use for soft tissue reconstruction of the hand, all free TPFF of the superficial temporalis fascia during the years 2007–2016 at our institution were analysed.

## Methods

Ethical approval for this study was obtained from the institutional ethic committee (26319_Bc). Between the years 2007–2016, 14 patients received a free TPFF of the superficial temporalis fascia for soft tissue reconstruction. The youngest patient was 17, the oldest 80 (mean age 53 years). The defect had an average size of 14.3 cm^2^.

### Operative procedure

Preoperatively, the STA was localized by Doppler examination. After incision in the hairline, the scalp flap including the subcutaneous fat was removed from the temporoparietal fascia. The STA and the superficial temporalis vein were exposed and followed until they reach the parotid gland. The temporal branch of the facial nerve was exposed as well and preserved. The dissection of the flap started from superior to inferior until the flap was dissected from the temporalis muscle and harvested as free flap. Finally, the flap was grafted with full-thickness or split-thickness skin.

The recipient vessels were exposed simultaneously to flap harvesting. All arterial anastomoses were performed end-to-side, for the venous anastomoses, a microvascular anastomotic coupler was used (Synovis MCA, Birmingham).

Beside age and gender of the patients, the defect size, entities of the defect and the defect region were analysed. Furthermore, the comorbidities of the patients as well as the length of the stay in hospital were recorded. Major complications were defined as flap necrosis and paresis of the facial nerve. Minor complications included haematoma or delayed wound healing without revision surgery.

The statistics were performed by using SPSS Version 24 (IBM, Armonk, USA). For unrelated, parametric test variables, the Student´s *t* test was used, for unrelated, non-parametric test variables, the brown-Mood-Median-test as well as the Mann–Whitney-*U*-test were used. The Kruskal–Wallis-Test was used in metric test variables.

## Results

**Fig. 1:**
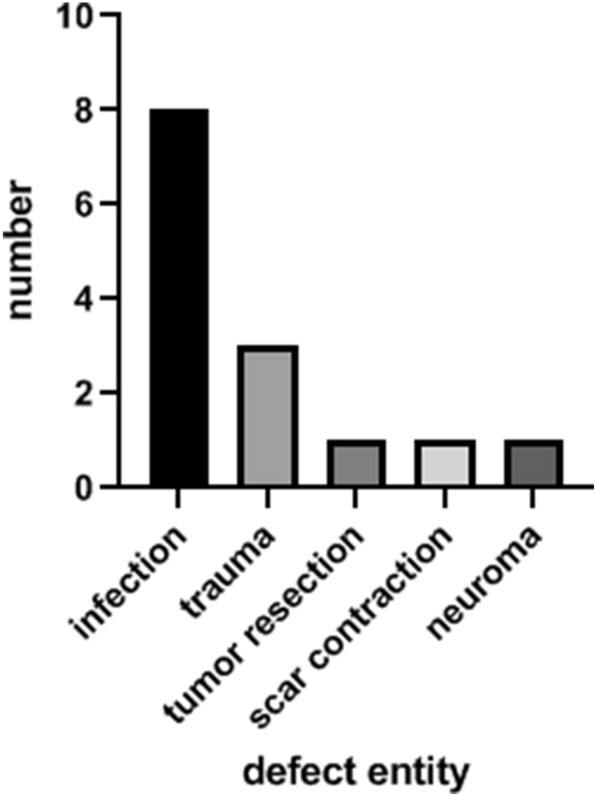
Defect entities

During 2007–2016, 14 patients received a free TPFF for soft tissue reconstruction of the upper extremity. Seven patients were males (50%), and seven were females (50%). There were five main entities (Fig. 1) for the defects presented in this study: infection (*n* = 8), trauma (*n* = 3), tumor resection (*n* = 1), scar contraction (*n* = 1) and neuroma (*n* = 1). The detected pathogens were staphylococcus aureus (*n* = 4), klebsiella pneumoniae (*n* = 1), actinomyces viscosus (*n* = 1), streptococcus pyogenes (*n* = 1) and neisseria weaveri (*n* = 1). Comorbidities were found in 71% of patients (*n* = 10). Most patients suffered from arterial hypertonia (*n* = 7), followed by diabetes mellitus (*n* = 3) and hypercholesterolemia (*n* = 2). Additional comorbidities were hyperuricemia, rheumatism, Factor V Leiden mutation, atrial fibrillation, hypothyreoidism, heart valve replacement and chronic heart failure. Mean follow-up period was 13 weeks (1–31 weeks). The mean defect size was 14 cm^2^ (11–20 cm^2^, ± 13 cm^2^). All free TPFF were transplanted to the upper extremity. Eight of them were used for reconstruction of a finger (57%), three for soft tissue reconstruction of the dorsum of the hand (22%), two for the palm of the hand (14%) and one for the forearm (7%).

The time required for surgery was 4 h 26 min (± 59 min); patients stayed in hospital after soft tissue reconstruction for 9.5 days.

**Fig. 2:**
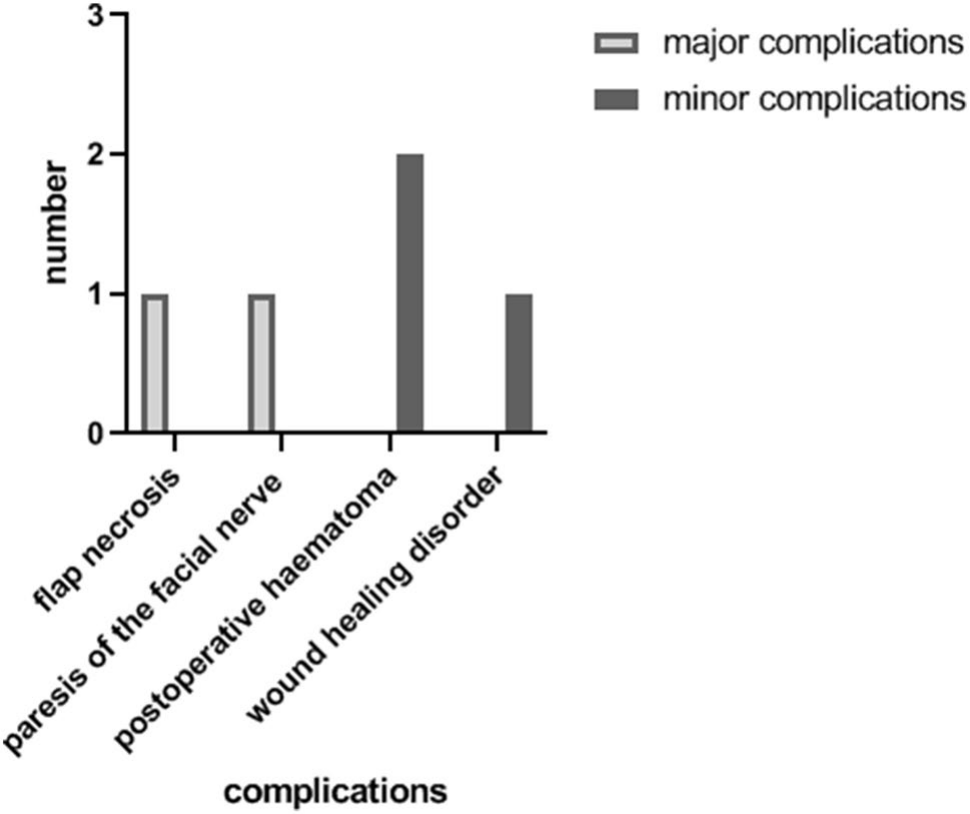
Major and minor complications

Complications were recorded in five patients (Fig. 2). In two cases of all TPFF (14.3%), major complications were found. There was one flap necrosis, so the patient received a free serratus muscle flap as second reconstruction. One patient suffered from paresis of the facial nerve with a paresis of the forehead and the eyebrow. Minor complications were found in three patients (21.4%): two patients suffered from postoperative haematoma. One patient had impairments of wound healing. Alopecia was found in none of the 14 patients.

The time required for the operation had no statistically significant influence on the postoperative complications.

Even though the postoperative stay in hospital was longer in patients of higher age, we did not find a statistical significance (*p* = 0.35). The number of comorbidities had no influence on the duration of the stay in hospital (*p* = 0.177) or on the number of complications (*p* = 0.39).

**Fig. 3:**
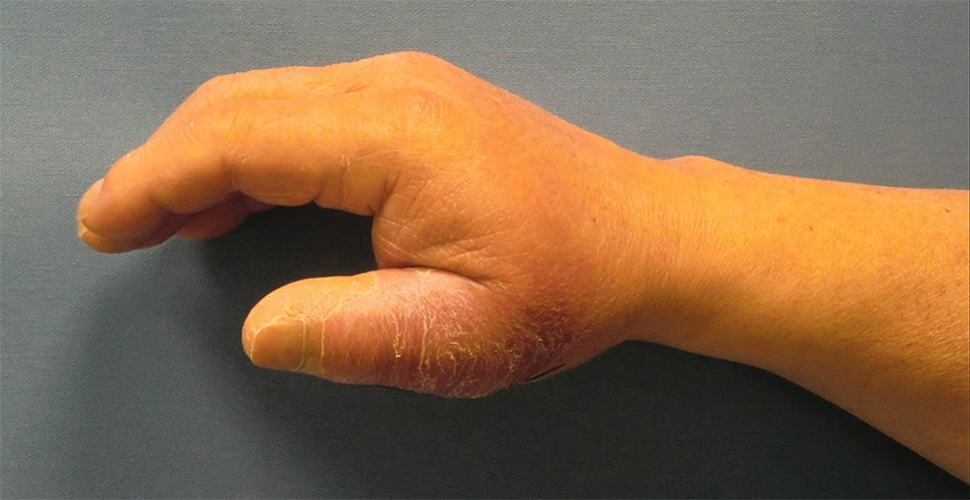
73-year-old male patient with an infection of his right thumb

**Fig. 4:**
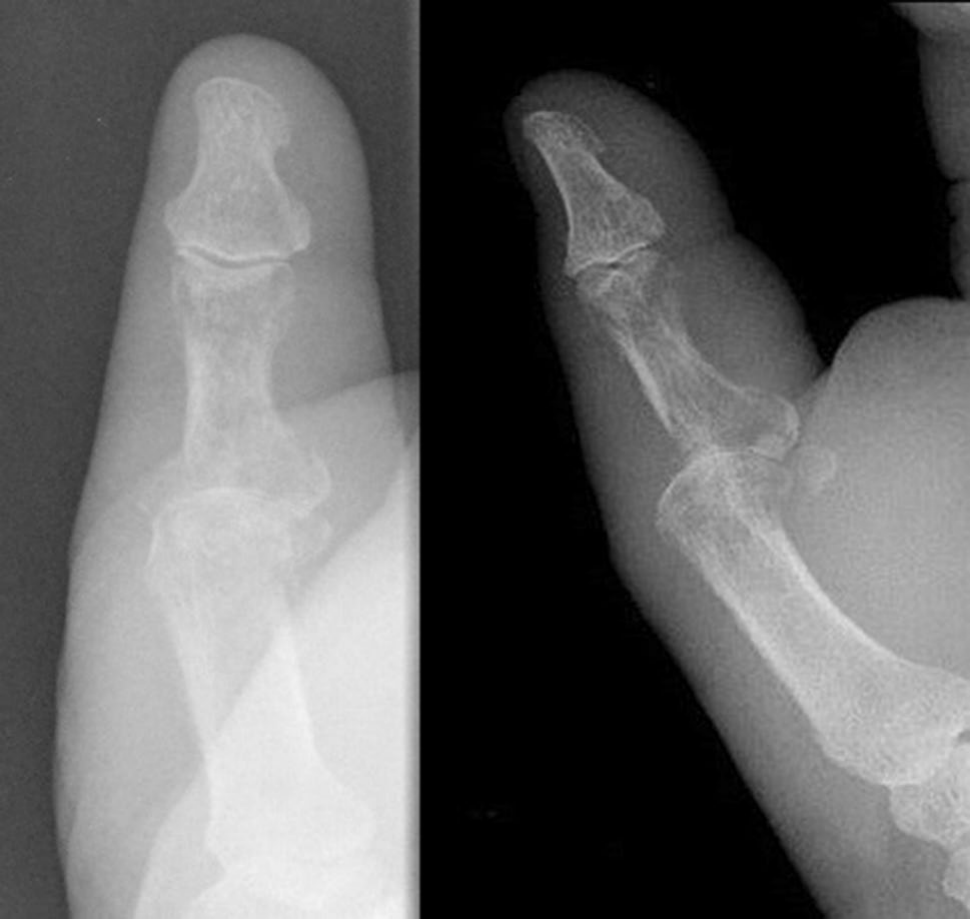
X-ray showing osteomyelitis of the proximal phalanx of the right thumb

**Fig. 5:**
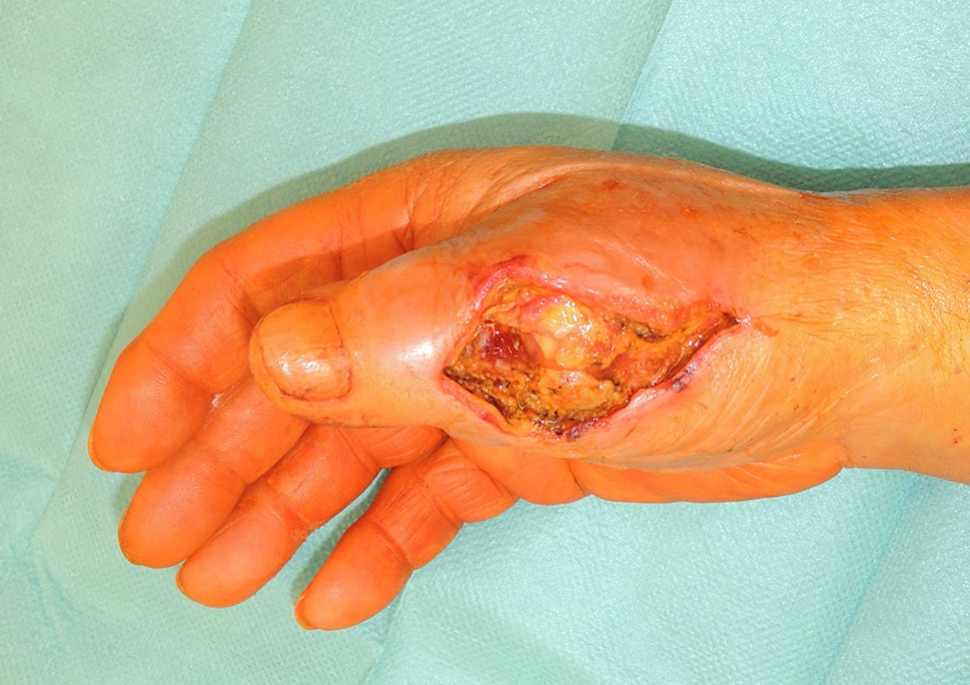
Debridement with partial bony resection

**Fig. 6:**
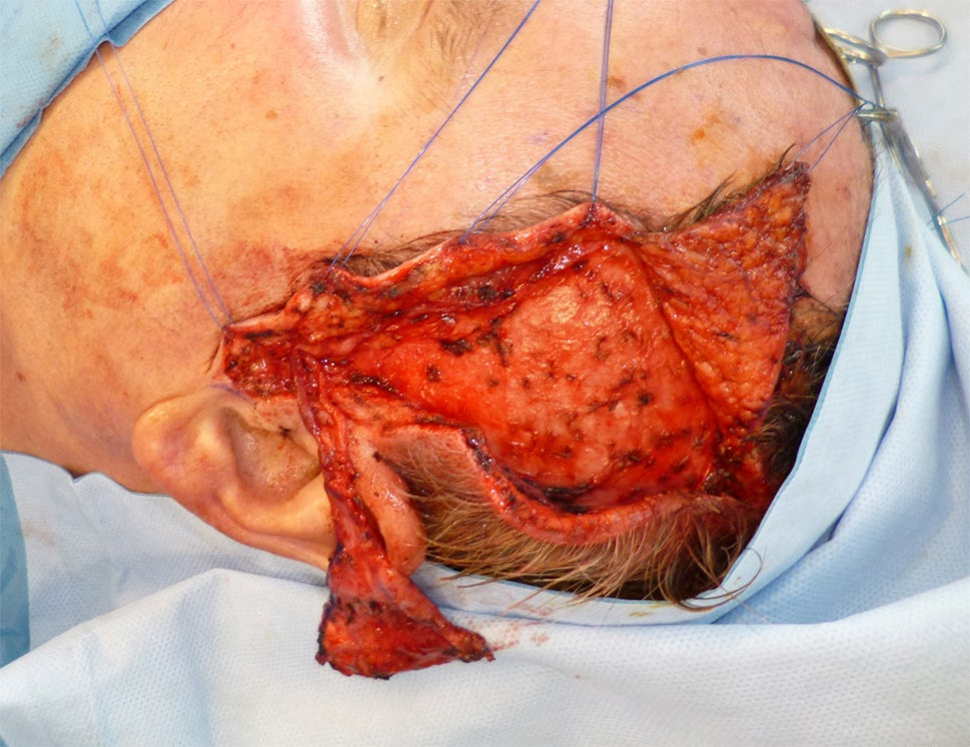
Tempororoparietal fascia flap after harvest

**Fig. 7:**
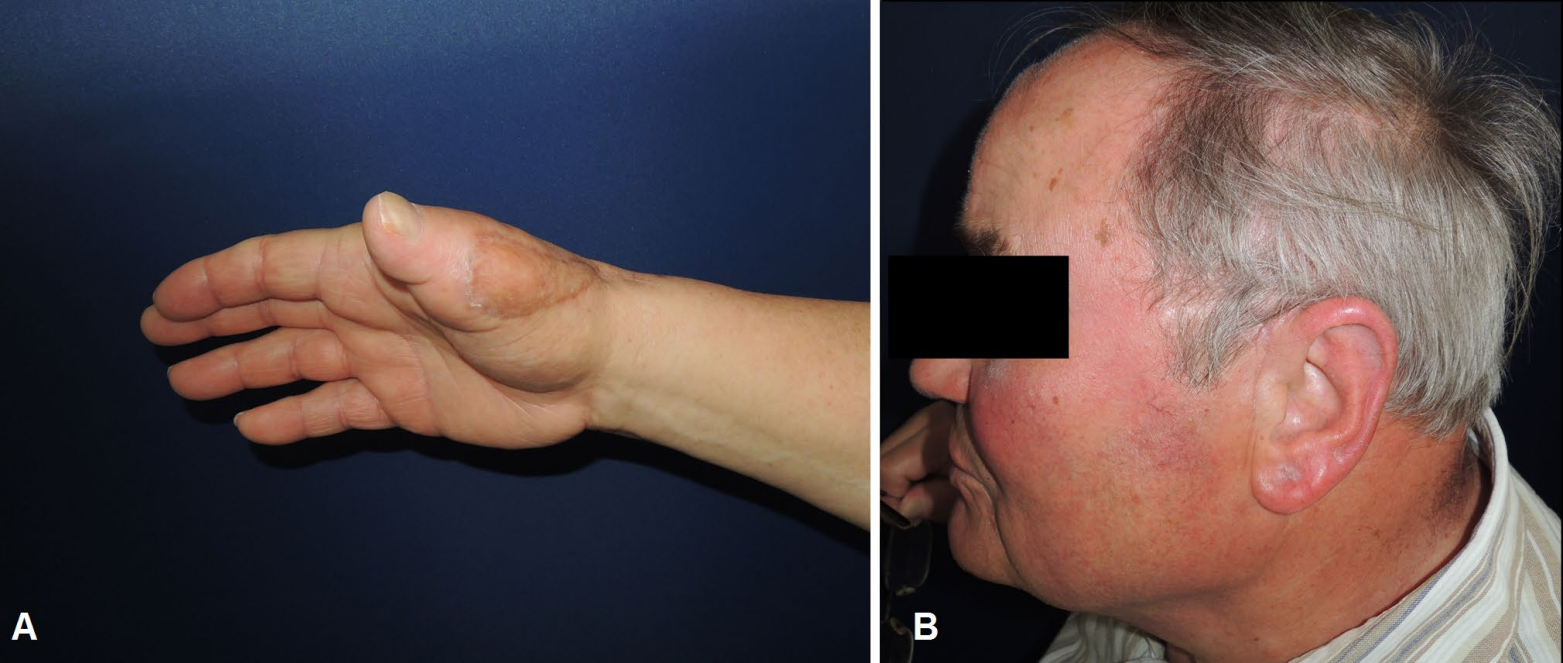
Results 3 months after free TPFF (**a**) with inconspicuous scar at the donor site (**b**)

**Fig. 8:**
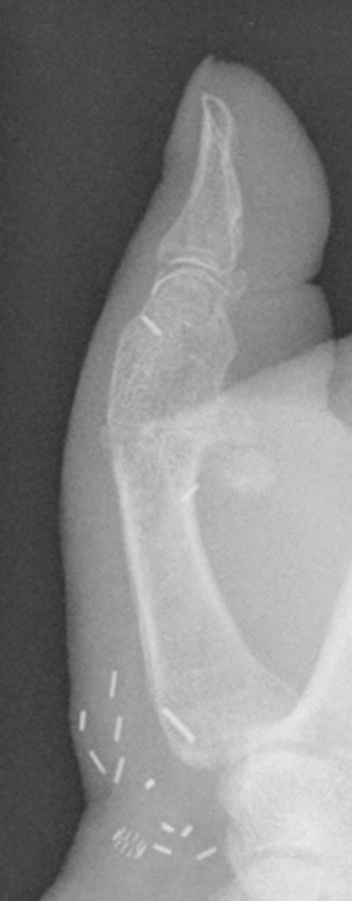
X-ray of the right thumb 7 months after the end of the antibiotic treatment

A 73-year-old male patient presented an infection of his right thumb (Fig. 3) with osteomyelitis of the proximal phalanx that was diagnosed previously with X-ray (Fig. 4) and an MRI. The patient received multiple debridements including partial resection of the infected bone (Fig. 5). Histomorphological examination of bone samples of the proximal phalanx showed an osteomyelitis. Local antibiotics (Septopal^®^) were applied as recommended treatment for bone infections [12]. Furthermore, he received antibiotics—first Amoxicillin Clavulanate followed by Clindamycin due to the development of an exanthema—to address the detected Staphylococcus aureus for a total of 6 weeks. The patient received a free TPFF for soft tissue reconstruction when the wound was macroscopically clean (Fig. 6). The radial artery and its accompanying vein were chosen as recipient vessels. A 3.0-mm microvascular anastomotic coupler was used for the venous anastomosis. Three months after soft tissue reconstruction (Fig. 7a), the scar of the donor side was inconspicuous (Fig. 7b). Four months after soft tissue reconstruction and after healing of the osteomyelitis, the bony reconstruction of the proximal phalanx of the right thumb was performed by transplanting an iliac crest bone graft including an arthrodesis of the first metacarpophalangeal joint,. Five months after bony reconstruction, the patient was able to oppose the thumb to the little finger with just 1 cm distance. Seven months after the end of the antibiotic treatment, the X-ray of the right thumb did not show a sign of osteomyelitis, a sufficient bony consolidation was seen (Fig. 8).

## Discussion

The TPFF is widely used in plastic and reconstructive surgery. The combination of the very thin temporalis fascia and a skin graft is useful when a very thin layer of tissue is needed—for example at the palm or instep region when tendons, bones or joints are exposed so a simple skin graft is not suitable [13]. The STA has a constant anatomy, making flap harvesting a straight forward procedure. In addition, the donor site morbidity is generally low. Since it offers a good gliding surface, the flap has been published for a variety of indications as a free or pedicled TPFF in reconstructive surgery at any part of the body Soft tissue reconstruction of the hand requires best possible functional outcome, scar contractures and wound healing disorders should be avoided [14].

The management of soft tissue defects requires a staged strategy. According to the reconstructive ladder, local flaps should be taken into consideration before choosing free flaps. So an alternative treatment for soft tissue defects of the hand and wrist is the reconstruction using reverse forearm flaps such as the reverse adipofascial radial forearm flap (RARFF). Its harvesting side usually needs to be closed with a skin graft in contrast to the one of the TPFF, where direct closure is possible. Furthermore, the RARFF requires sacrificing one major artery of the arm [15]. Modifications of the flap based on a perforator of the radial artery have already been described, but they result in longer scars and might have higher complication rates compared to axial flaps because of the weaker plexus perfusion [16, 17]. Beside the RARFF, a pedicled flap based on the distal cutaneous branch of the ulnar artery might be an adequate treatment option [18]. The main advantage of those reverse forearm flaps is the possibility of soft tissue reconstruction of the hand using a pedicled flap, that usually results in a shorter duration of the operation. Microsurgical risks such as thrombosis of the vessels ending in flap loss should be reduced. Nevertheless, partial flap necrosis up to one-third of the flap area due to venous congestion was reported in 8.9% in patients with a pedicled flap based on the distal cutaneous branch of the ulnar artery [18]. In addition, partial flap loss is one of the main complications in RARFF. Rogachefsky et al. reported partial flap loss in 16.7% of cases [19]. Others found partial flap loss or epidermiolysis of the whole flap in 4.7% of RARFF [20]. The rates of partial flap loss are even higher in flaps based on a perforator of the radial artery, where partial flap loss was reported in two-thirds of all cases [21].

Free TPFF are mostly used in soft tissue reconstruction of the upper or lower extremity as after avulsion injury to both hands with exposed bones and tendons [22]. Or after trauma (burns, radiation, gunshot) or after replantation of a finger [23]. Furthermore, the free TPFF was used for augmentation of the first web space in 13 patients with ulnar nerve palsy—in total 14 TPFF with one patient having bilateral correction of the first web space [9]. In contrast to these studies, we present five different soft tissue defect entities. In our study, 14 free TPFF were transplanted to the upper extremity, after infection, trauma, scar correction, tumour resection or neuroma.

As in other types of flaps, the main complications of transplanting a free TPFF are flap necrosis, haematoma, seroma and delayed wound healing [24]. The complications are similar in studies reporting transplantation of the free TPFF. Yang et al. found in 11 free TPFF for soft tissue reconstruction of the lower extremity two major complications with venous crisis and one skin necoris. No flap necrosis and no donor site morbidity were found [13]. Woods et al. reported a flap survival rate of 95% (21 flaps) at the lower extremity, which is similar to our results. Partial flap necrosis was found in four patients. Four patients suffered from transient alopecia at the donor site, one patient had permanent alopecia. Temporary palsy in the forehead as injury to the temporal branch of the facial nerve was found in one patient [25]. We have recently utilized the near-infrared Indocyanine-green (ICG) fluorescence method intraoperatively to determine the true extend of the vascular supply within the whole flap and have successfully tailored the flaps according to the ICG perfusion mapping which allows us to discard any potentially non vital flap parts and to avoid necrosis of the flap margin [26–28].

The mean age of the patients in our study (53 years) was higher compared to other studies showing that reconstruction is possible even at higher age [9, 25]. The complication rate in our study was similar to others: major complications occurred in 14.3%, minor complications were found in 21.4%. There was one total flap necrosis of a free TPFF requiring additional surgery due to venous insufficiency that could not be solved by revision as in other types of flaps [29], so the overall flap survival rate was in total 93%. Temporary paresis of the facial nerve was found in one patient (7.1%). The major and minor complications in our study were flap necrosis, haematoma and disorders in wound healing as well as the donor site related complications of the temporal region as paresis of the facial nerve [30]. Both the high flap survival rate as well as the quite low donor side morbidity with a hidden scar resulting in a good aesthetic outcome show that the soft tissue reconstruction of the distal upper extremity with a free TPFF might be equally or even superior to the one with pedicled flaps of the ulnar or radial arterial systems.

According to the literature, the most often noted donor site morbidity is transient or permanent alopecia [7], why an endoscopic surgical harvest of the flap was invented to reduce the risk of alopecia [31]. We did not observe alopecia in any of the 14 patients of our study.

The mean follow-up period (13 weeks) in our study was shorter compared to other studies with follow-up periods up to 54 months [25] or 64 months [9]. A reason might be that many patients live far away from our hospital and only came to our hospital to receive the operative therapy. This may result in the postoperative care being carried out in facilities close to their home.

The venous coupler was used because of the reduced post-operative thrombosis risk and the shorter ischaemia time compared to hand-sewn venous anastomosis [32, 33].

We concentrated on single-stage reconstruction of soft tissue defects thus having a less complicated surgical procedure compared to complex reconstructions including bony defects or multiple stage reconstructions. Unfortunately, more specific results of the functional and aesthetic outcome were not possible due to the retrospective character of the study. We presented soft tissue reconstruction of the hand and upper extremity after different defect entities showing the versatile use of the free TPFF.

## Conclusion

The TPFF is a versatile and useful tool in plastic-reconstructive surgery. As a free flap, it can cover soft tissue defects all over the body whenever a very thin layer of vascularized tissue is needed, especially in the distal upper extremity. Beside its constant anatomy, it has an inconspicuous donor-site morbidity. The complication rate we presented as major and minor complications is moderate.
